# English Sanitary Institutions

**Published:** 1899-06

**Authors:** 


**English Sanitary Institutions.** By Sir JOHN SIMON, K.C.B. Second Edition. Pp. xix., 516. London: Smith, Elder, & Co. 1897.

We gladly renew acquaintance in this second edition of Sir John Simon's *English Sanitary Institutions* with this fascinating and instructive history of human sanitary endeavour. The successful writer on matters of universal history must be a man of wide knowledge and of broad mind, able to sift and choose, to prune and criticise, and to weave the disconnected threads of record and tradition into one continuous whole. Sir John Simon has the necessary qualities for the work, and it is fitting that from the pen of so delightful a writer such a volume should appear after a long life of usefulness, happily not yet completed.

The introduction takes us back to "that nebula of times which human records do not pretend to reach," and presupposes that at a very early period when communities began to exist, a desire must have originated "to amend, in the circumstances common to them, the conditions which they found dangerous to their lives." Hence through succeeding phases of civilisation, from the evidence found amid the ruins of Nineveh, from the records relating to Greece, and through the more pretentious evidences such as the aqueducts and cloacæ of Rome, and the establishment in Rome of a public medical service for the poor, we pass through the darker centuries, the crusades, and the spread of leprosy, and the widespread and disastrous epidemics of the Middle Ages. The efforts of medieval philanthropy to relieve emergencies of distress threatening the poor are duly recognised, and the early and tentative efforts to cope with disease are in turn pointed out. Then it is shown that through the Tudor and Stuart reigns the changes developing later into the beginnings of modern medicine had been tending to define themselves in embryo; and medical science, previously "exercised from the widely different standpoints of the ecclesiastic, the barber, and the grocer," was able to "come into self-conscious existence."

In "New Momenta" we trace the beginnings of modern medicine, accompanying and following the revival of learning in Europe, starting from the fifteenth century, at first vaguely, more definite in the sixteenth century when alchemy made progress in experiments and Vesalius achieved his daring dissections; through the more eventful quickening of the art of medicine in the seventeenth century, signalised in its earlier half by Harvey's discovery of the circulation of the blood, and later by Sydenham's careful and exact observations; leading to the far higher position of the practice of medicine in the eighteenth century, and its still more illustrious development in the present. Briefly, but with due appreciation, are sketched the work of Mead, whose *Short Discourse concerning Pestilential Contagion* gave advice wonderfully in advance of his age, and might even now repay study; Pringle and Lind's work on the Diseases of the Soldier and Sailor; Gilbert Blane's work in banishing scurvy from the navy; George Baker's work on the Cider Colic in Devonshire; and Jenner's imperishable triumph, whose "services to mankind, in respect of the saving of life, have been such that no other man in the history of the world has ever been within measurable distance of him."

And so we pass in chapters of unabated charm through the history of "The Growth of Humanity in British Politics," wherein John Howard's memorable work on *Gaols* is not forgotten; touch on the early experiences of Asiatic cholera in Europe, and reach the commencement of the present reign. Here Sir John Simon is thoroughly at home. Stimulating as it was to trace with him the inception, early beginnings, and gradual growth of preventive medicine, it is still more deeply interesting and instructive to follow under the guidance of his master-hand, and with the sidelight of his ripe experience, the actual growth of the great revival in the care of health which commenced with the reign of Queen Victoria and has progressed steadily, with occasional relapses, but in the main ever making for improvement and for progress. The volume must be read and studied to fully appreciate all the author's conclusions; but it may be stated that, in his belief, "the educational onward impulse" towards development of our sanitary institutions and their administration must come, as it has largely come in the past, from the medical profession. He looks forward with hopefulness, gladdened by "the immense development of altruism," by the "kindliness of man to man," and sees the human race "more and more, as the cycles are trodden," rising "to the religion of mutual helpfulness."

